# Pullulan based derivatives: synthesis, enhanced physicochemical properties, and applications

**DOI:** 10.1080/10717544.2022.2144544

**Published:** 2022-11-11

**Authors:** Surendra Agrawal, Divya Budhwani, Pravina Gurjar, Darshan Telange, Vijay Lambole

**Affiliations:** a Department of Pharmaceutical Chemistry, Datta Meghe College of Pharmacy, Datta Meghe Institute of Higher Education and Research (DU), Sawangi Meghe, Wardha, India; b Department of Industrial Pharmacy, Shobhaben Pratapbhai Patel School of Pharmacy & Technology Management, Mumbai, SVKM’S NMIMS, Mumbai, India; c Department of Pharmaceutics, Sharadchandra Pawar College of Pharmacy, Otur, Pune, India; d Department of Pharmaceutics, Datta Meghe College of Pharmacy, Datta Meghe Institute of Higher Education and Research (DU), Sawangi Meghe, Wardha, India; e Department of Pharmacology, Datta Meghe College of Pharmacy, Datta Meghe Institute of Higher Education and Research (DU), Sawangi Meghe, Wardha, India

**Keywords:** Pullulan derivatives in drug development, pullulan in drug delivery optimization, properties of pullulan derivatives, pullulan complex in drug delivery

## Abstract

Drug distribution relies heavily on polymers, which also offer a variety of benefits like controlled release, targeted release, prolonged release, etc. Due to their low toxicity and great safety, biodegradable polymers are highly preferred. The exopolysaccharide known as pullulan is generated from a fungus known as *Aureobasidium pullulan*. It has many different qualities, including biodegradability, appropriate adhesion, antioxidant, film-forming capacity, blood compatibility, mucosal adhesion, etc. However, its application in the pharmaceutical industry is restricted by its insolubility in organic solvents, mechanical characteristics, and lack of macromolecule-carrying ability groups. This review provides an overview of the modifications made to pullulan, including periodate oxidation, etherification, esterification, sulfation, urethane derivatization, PEG incorporation, and cationization, to enhance its solubility in organic solvents, mechanical properties, pH sensitivity, drug delivery, anticoagulant, and antimicrobial properties. Pullulan has nine active hydroxyl groups in its structure that react chemically that can be used for physicochemical modification to produce pullulan derivatives. A key area of pullulan research has been pullulan modification, which has demonstrated enhanced solubility, pH-sensitive targeting, broadened horizons for delivery systems, anticoagulation, and antibacterial properties.

## Introduction

Polymers are interesting material because of their unique characteristic and wide application (de Carvalho et al., 2020). They are frequently used to deliver medications. They can create a membrane or matrix to regulate a drug’s release over a longer duration, which can assist lessen the need for frequent doses. In addition, they can be utilized for the nanodrug delivery of insoluble or biotechnology-based medications. Another potential with polymer is targeting a certain delivery. Active pharmaceutical ingredient (API) is often released from polymers through diffusion, degradation, or disorganization. Although there are many different kinds of polymers, biodegradable polymers are strongly advised. For this reason, the formulations’ building blocks include polysaccharides, phospholipids, and polypeptides (Riva et al., 2011).

Due to their distinct physical and chemical characteristics, polysaccharides may find use in industry. Due to its remarkable adaptability and flexibility, the polysaccharide pullulan, which was initially discovered in 1958 in a strain of the fungus Aureobasidium, is frequently employed in the pharmaceutical business (Singh et al., 2008). Apart from *Aureobasidium pullulan*, some other strains can also produce pullulan. Those strains are *A. melanogenum, A. mousonni., Fungus Cytaria harioti, Cytaria darwinii, Termella mesenterica, Cryphonectria parasitical, Teloschistes falvicans, Rhodotorula bacarum, Cryphonectria parasitica, Fungi Eurotium cheyalieri, E. cheyalieri, Aspergillus japonicus* and *Fungi Rhodosporidium paludigenum*, but *Aureobasidium pullulan* is preferred over other stains for large-scale production because of its superior quality and high production rate (Raychaudhuri et al., 2020).

Pullulan is a highly expensive polymer due to its high production charges. It has been supplied by Hayashibara Company Limited in Okayama, Japan, since 1976 (Singh et al., 2008). Its molecular weight varies from 4.5 × 10^4^ to 6 × 10^5^ Da (Pobiega et al., 2020). It has a high glass transition temperature, which helps in protein stabilization in high humid conditions (Tian et al., 2018). It appears as white or yellowish-white powder, with 5 to 7 pH. Pullulanase is an enzyme that hydrolyzes the pullulan (Ganie et al., 2020). There are two types of pullulanase, i.e. type I and type II. Type I pullulanase acts on α-(1,6) glycosidic bond to produce maltotriose unit, while type II pullulanase acts on α-(1,6) glycosidic bond and α-(1,4) glycosidic bond to produce maltotriose and mixture of glucose, mannose, and maltotriose, respectively. Other enzymes such as pullulan hydrolase type I (neopullulanase) and pullulan hydrolase type II (isopullulanase) acts on α-(1,4) glycosidic bond to produce panose and isopanose, respectively (Hii et al., 2012).

Pullulan is a water-soluble extracellular polysaccharide that can be used for film formation. Still, due to its highly water-soluble nature, the formed film will get washed off when it comes in contact with water (Coltelli et al., 2020; Pobiega et al., 2020). The derivatization of pullulan can impart hydrophobicity that is required for good film (Singh et al., 2015). This formed film can be used as a food packaging material as it is biodegradable, nontoxic, odorless, tasteless, colorless, and antioxidant. Another issue with pullulan film is its brittleness after drying, which can be resolved by adding suitable plasticizers such as fatty acid, glycerol, and protein (Zhang et al., 2019; Coltelli et al., 2020).

Pullulan is preferred in the food sector as a low-calorie food ingredient in the production of low-calorie goods, for instance, synthetic rice, low-calorie noodles, baked foods, etc. Pullulan can be employed as a binder since it has adequate adhesive qualities (Yang et al., 2020). It is relatively low viscous compared to other polysaccharides and remains unaffected over a wide pH range, i.e. 2 to 11 pH (Prajapati et al., 2013). Beyond this temperature, it starts to decompose. It is thermostable up to a maximum of 250–280 °C. In addition, its lack of immunogenicity, lack of carcinogenicity, and lack of mutagenicity make it a polymer of choice for formulators and researchers to use as a carrier in targeted medication delivery and gene transfer (Singh et al., 2017).

However, few properties of pullulan such as poor mechanical property, insolubility in organic solvent, high cost of production, absence of hydrophobic group, limits the application of pullulan in various fields. Despite of having all these limitation, chemical derivatization and polymer modification have shown great success to further widen its application in pharmaceutical product development (Bruneel & Schacht, 1993; Shibata et al., 2001).

**Figure 1.**
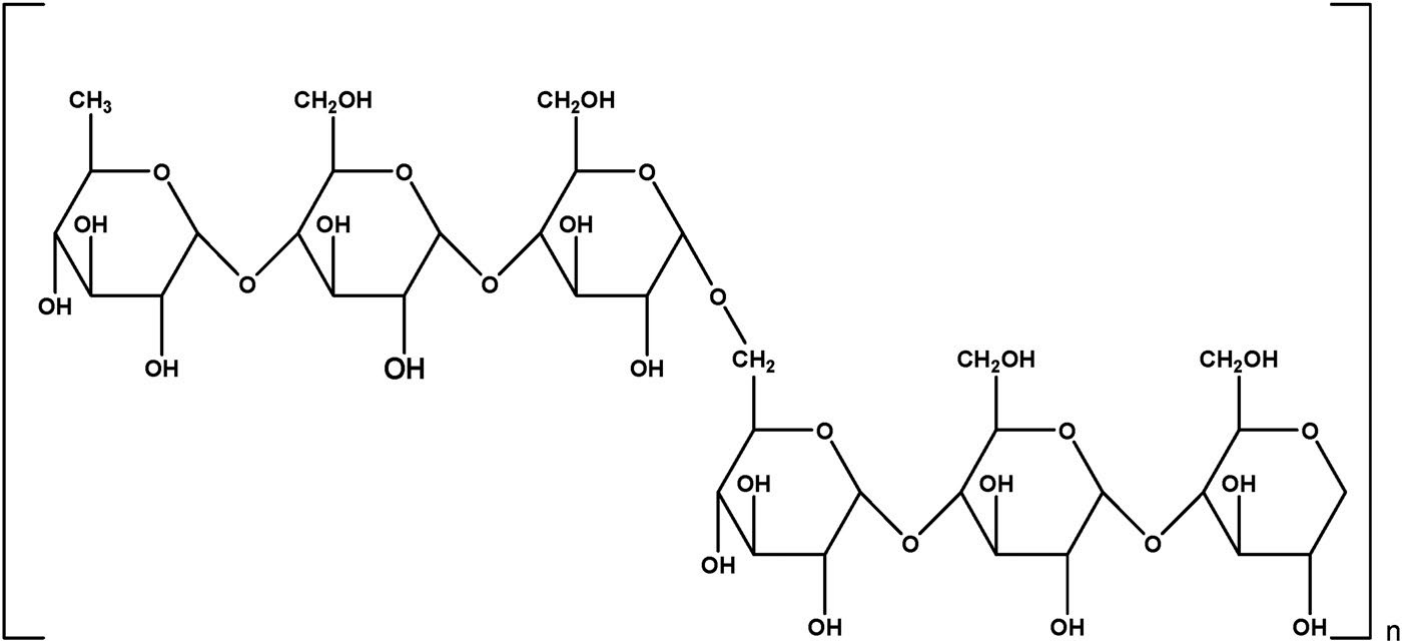
Structure of pullulan.

As shown in Figure 1, pullulan (C_6_H_10_O_5_)*_n_* consists of repeated units of maltotriose. Each pyranose ring of maltotriose is connected via α-(1,4) glycosidic bond, and one maltotriose unit is connected to another maltotriose unit via α-(1,6) glycosidic bond (Coltelli et al., 2020). Each maltotriose unit has nine OH groups, which can be replaced by groups with different functionality. Chemical modification improves the physical and chemical properties of pullulan, retains the unique properties of pullulan, and expands the application range. Modified pullulan have better mechanical strength, solubility in the organic solvents, and ability to deliver the drug at target site (Supe & Takudage, 2021). This review mainly focuses on the mechanism of pullulan derivatization and applications of pullulan derivatives due to enhanced property.

## Chemical modification of pullulan

Modification of pullulan is required to combine the benefits of natural polymer with other substances so that we can get a single compound with improved physicochemical properties.

Pullulan structure consists of repeated maltotriose units. Each maltotriose unit has nine hydroxyl groups. Modification can be done by introducing a new functional group in place of the hydroxyl group via oxidation, esterification, etherification, urethane derivation, sulfation, amination, etc. as illustrated in Table 1. Chemical modification improves physical and chemical properties as well as expands its application (Xu et al., 2019).

**Table 1.** Different types of reactions for chemical modification of pullulan.

Type of reaction		Substitute	Improved property
Periodate oxidation (glycosidic ring-opening)		Aldehyde	Mechanical property, pH sensitivity, drug delivery
Esterification	Succinylation	Carboxylic group	Stability
	Cholesterol modification	Cholesterol	Stability, drug delivery
Etherification	Carboxymethylation	Carboxymethyl	Mechanical property, antimicrobial property, drug delivery
	Alkylation	Alkyl group	Solubility in organic solvents
Urethane derivative		*N*-phenylurethane or *N*-hexylurethane groups	Solubility in organic solvents, Stability
Sulfation		SO_3_	Stability, anticoagulant property, drug delivery
Cationization		DBAP	pH sensitivity, drug delivery
PEG incorporation		PEG	Solubility in organic solvents

### Pullulan derivative for enhanced solubility

As a water-soluble polymer, pullulan has a wide range of applications, but its insolubility in organic solvent limits its application. Solubility enhancement in the organic solvent and water resistance is required to prevent film or coating washability in water. At present, there are generally three methods for solubility enhancement of pullulan in organic solvent: (1) alkylation of pullulan, (2) formation of urethane derivative of pullulan, and (3) incorporation of PEG in pullulan.

Alkylation involves the reaction of pullulan to form alkyl pullulan. It can be synthesized by the pullulan reaction with haloalkane in the presence of NaOH. The degree of substitution of haloalkane is inversely proportional to the glass transition temperature of pullulan, which means the glass transition temperature of pullulan decreases with an increase in the degree of substitution of pullulan. In contrast, an increase in the degree of substitution of haloalkane enhances the solubility in general organic solvents. Alkylated pullulan was prepared using propyl- and butyl-etherified pullulans (PrPL and BuPL), and shows improved solubility in a general organic solvent and resistance from the water (Shibata et al., 2002). Alkyl pullulan has application in modifying substances containing amine, hydroxyl, carboxyl, thiol, or any other group (Mocanu et al., 1999).

Formation of urethane derivative results in enhanced solubility of pullulan in general organic solvent, resistance from water, and thermostability (Zia et al., 2015). It can be prepared by the reaction of pullulan with phenyl isocyanate (PIC) or hexyl isocyanate (HIC), resulting in the introduction of *N*-phenyl urethane or *N*-hexyl urethane groups in the pullulan structure. The degree of addition of PIC and HIC alters the various properties of pullulan, such as an increase in its degree of addition, decreases the glass transition temperature and tensile strength of pullulan. The addition of PI to pullulan increases its solubility in ethanol, while the addition of HI to pullulan will increase its solubility in acetone and water (Shibata et al., 2001).

The incorporation of PEG in pullulan is another way to improve the solubility of pullulan in organic solvents. Pullulan-PEG is prepared by reaction of pullulan with *N*,*N* dimethylaminopyridine, PEG, carboxylic acid, and *N*,*N*-dicyclohexylcarbodiimide in the presence of DMSO. This combination results in enhanced solubility of pullulan in the organic solvent. PEG is selected for enhancing solubility because of its outstanding physicochemical property including hydrophilicity, solubility in organic solvents, lack of toxicity, and absence of antigenicity and immunogenicity. PEG incorporated pullulan may self-associate into micelles because PEG is soluble in organic solvent and pullulan is soluble in water. These micelles have unique sizes and good biocompatibility with blood, body fluid, and tissues, making them suitable for biomedical applications (Jiao et al., 2004).

### Pullulan derivative for improved mechanical property

Mechanical property in a polymer is required to retain its properties under stress. Pullulan is a brilliant film former, but films prepared from this polymer are brittle in nature due to its poor mechanical property. Carboxymethylation and periodate oxidation of pullulan can help to enhance its performance by improving the mechanical property of pullulan.

**Figure 2.**
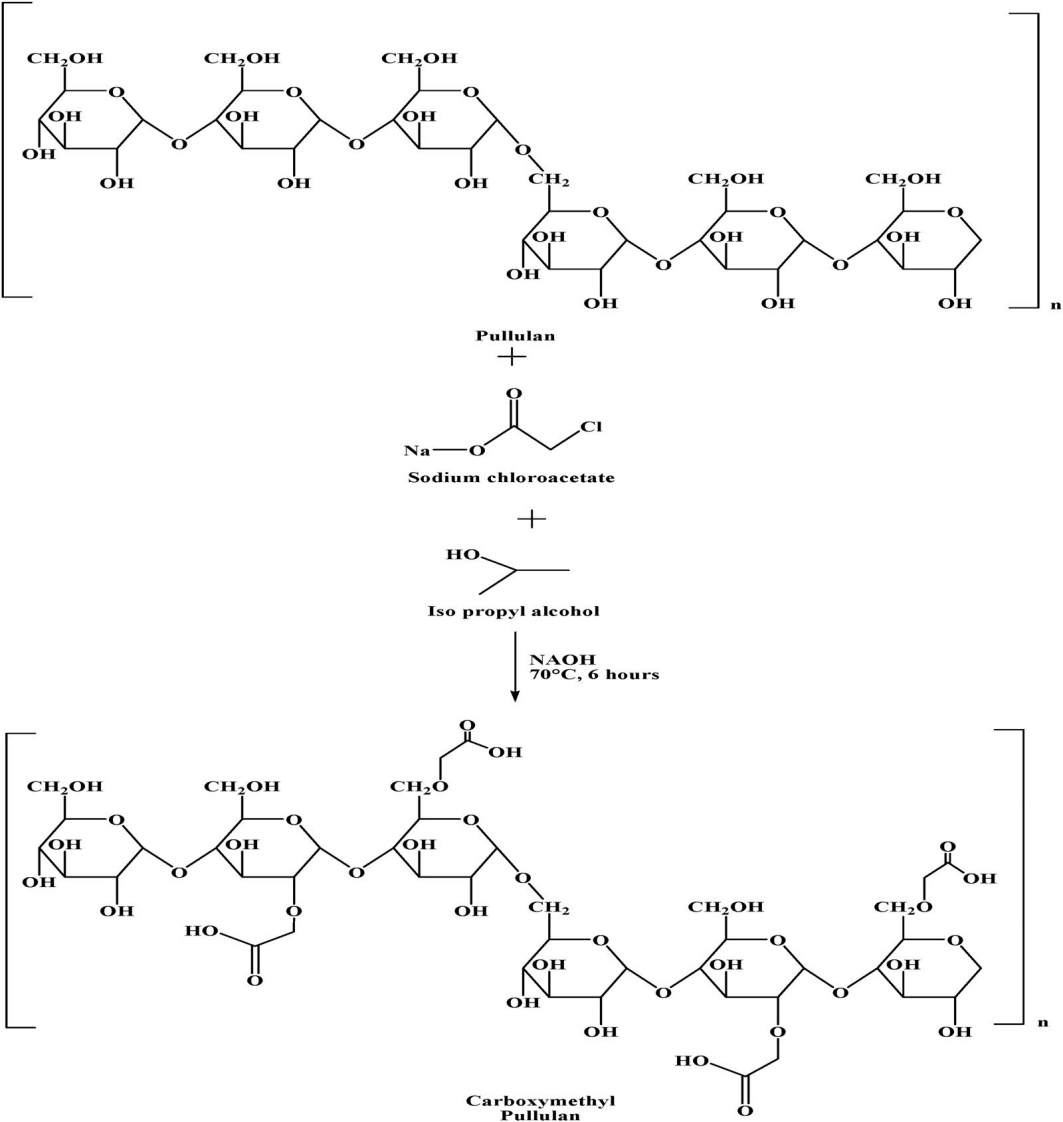
Scheme for synthesis of carboxymethyl pullulan.

Carboxymethylation involves the introduction of carboxylate or carboxymethyl chitosan into pullulan structure. Carboxymethylated pullulan can be prepared by a reaction of pullulan with sodium chloroacetate and isopropyl alcohol as shown in Figure 2. It results in the introduction of carboxylate groups in the structure of pullulan (Xie et al., 2021). Carboxylate pullulan can be used for targeted drug delivery as it becomes pH and ionic strength sensitive (Singh et al., 2015). Apart from carboxylate pullulan, pullulan-carboxymethyl chitosan can also be prepared via carboxymethylation mechanism. This is done by the introduction of carboxymethyl chitosan into pullulan. Formation of pullulan-carboxymethyl chitosan is required to overcome the aforementioned listed issues associated with pullulan film. Also, pullulan in pure form is quite expensive, limiting the use of pullulan. So, modification with the substance having good mechanical properties and low costs will overcome all these limitations. Chitosan is cationic biopolymer with desired mechanical properties with additional features like biodegradability, biocompatibility, and low oxygen permeability. Carboxymethyl chitosan (CMCH) is a water-soluble derivative of chitosan, which can be conjugated with pullulan to form carboxymethyl pullulan (CMP). The resultant derivative i.e., CMP not only enhanced the mechanical property of film but also reduced the cost of the compound. In addition, carboxymethyl chitosan has antimicrobial property so the introduction of CMCH in pullulan forms films with antimicrobial property (Wu et al., 2013).

An edible film was prepared using carboxymethyl chitosan-pullulan for preserving mangoes. The film resulted in the prolonged shelf life of mangoes. In addition, Galangal essential oil (GEO) was added to get antimicrobial action. GEO has antioxidant properties along with antitumor, anti-inflammatory, and anticoagulation action. Carboxymethyl chitosan-pullulan is a promising food packaging agent to prolong the shelf life of fresh and processed products (Zhou et al., 2021). An injectable hydrogel of amoxicillin was obtained by grafting poloxamer 407 in CMP. The hydrogel showed great elasticity and excellent ability to recover the initial structure after the removal of applied stress. Poloxamer 407 is a thermosensitive copolymer. This combination showed sustained release of amoxicillin, which indicates the success of CMP-poloxamer copolymer for the formulation of injectable hydrogels (Constantin et al., 2021).

Periodate oxidation of pullulan, also enhances the mechanical strength. Many polysaccharides such as dextran, starch, and cellulose derivatives are being used to carry the macromolecular prodrug. Pullulan has been selected as a carrier because it has blood compatibility and biodegradation property. However, the pullulan structure does not have any functional group that can carry the macromolecular product. Periodate oxidation of pullulan refers to the reaction of pullulan with sodium periodate to introduce aldehyde group in pullulan. Introduction of the aldehyde group results in direct coupling of polysaccharide with the drug which is required to be a suitable carrier (Bruneel & Schacht, 1993). A gelatin hydrogel was prepared using periodate oxidated pullulan. This resolved the poor crosslinking of gelatin and resulted in the enhanced compressive strength of gelatin hydrogel due to enhanced crosslinking, which requires the high stress to break the covalent bond between aldehyde group of modified pullulan and amine group of gelatin (Wang et al., 2019).

### Pullulan derivative for enhanced stability

Stability is an essential attribute for any product. Stabilizing agents are added to the product to prevent it from degradation. Pullulan can also be used as stabilizing agent on modification via succinylation, cholesterol incorporation, and urethane derivatization.

**Figure 3.**
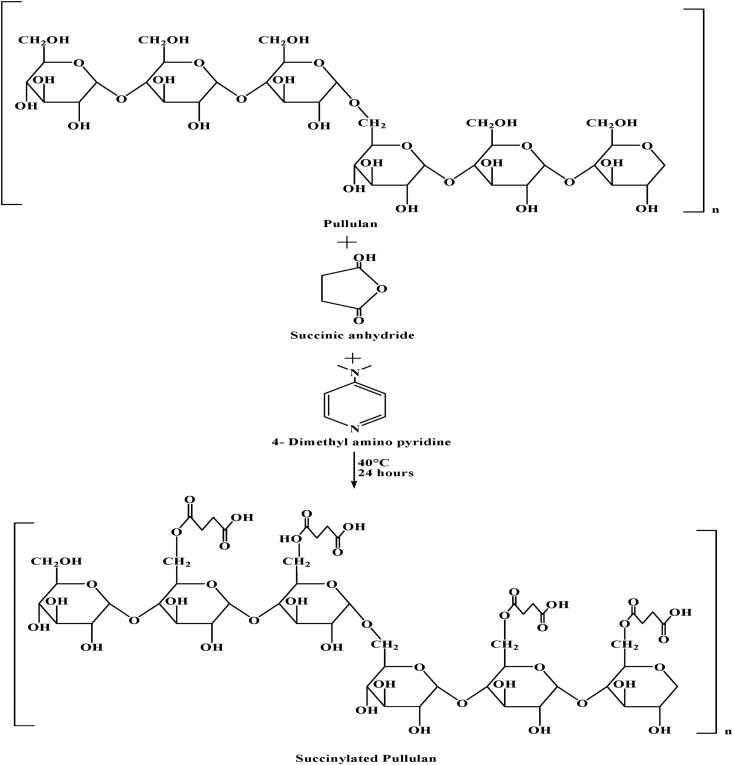
Scheme for synthesis of succinylated pullulan.

Pullulan is a highly biocompatible and biodegradable polymer that can be used as a carrier to deliver macromolecules. It is a water-soluble polymer with hydrophilic properties, making it difficult to encapsulate hydrophobic and charged protein (Bruneel & Schacht, 1994). To overcome this issue, hydrophobic or charged segments were introduced in pullulan. Succinylation results in the incorporation of the carboxylic group into pullulan [Pullulan acetate (PLAc), pullulan propionate (PLPr), and pullulan butylate (PLBu)] by reaction with negatively charged succinic anhydride (acetic anhydride, propionic anhydride, and butyric anhydride) that makes it appropriate for drug delivery of positively charged protein (Niu et al., 2019). As shown in Figure 3, succinylation occurs in the presence of catalyst DMSO (4-dimethylaminopyridine) at 40 °C for 24 h. The preferred site of succinic anhydride in pullulan is C-6. Succinylated pullulan requires activation of COOH group by *N*,*N*′-carbonyldiimidazol. The resulting derivative can be coupled with the amine (Bruneel & Schacht, 1994). A microsphere was prepared using SPA (succinylated pullulan acetate) to carry protein. In the microsphere, PLGA [poly(dl-lactic acid-*co*-glycolic acid)] was replaced with SPA and loaded with lysozyme (Lys) as a model protein drug using the double emulsion method. This microsphere resulted in long-term protein stability and three times higher protein loading efficiency. Therefore, protein can be delivered with long-term stability using SPA (Woo et al., 2011). A film to extend the shelf life of fruit was also prepared. They packed strawberries with pullulan acetate film, which showed reduced weight loss percentage and enhanced shelf life of strawberries with its antioxidant property and by exhibiting high water contact angle. This indicated the pullulan as a promising material for the edible coating to extend the shelf life (Niu et al., 2019).

Cholesterol modification of pullulan is one more way to enhance the stability of formulation. Cholesterol is incorporated into pullulan as a hydrophobic group. A self-assembled nanoparticle of Epirubicin was prepared using cholesterol-modified pullulan. The nanoparticle showed enhanced drug stability with a long half-life, higher blood plasma concentration, and comparatively less drug toxicity (Yuan et al., 2020). Another nanoparticle using cholesterol-modified pullulan was prepared to load the mitoxantrone (MTO) drug. The selected drug has nonselective for tumors thus, has many side effects. Loading MTO in cholesterol-modified pullulan nanoparticles resulted in targeted drug delivery and enhanced stability, permeability, retention effect, efficacy, and reduced drug update by normal tissues. Further addition of human serum albumin (HAS) in MTO-CHP nanoparticle better stabilized the drug release by slowing down drug release rate in acidic media (Yuan et al., 2019).

Apart from the above two modifications, the urethane derivative of pullulan also aims to enhance the thermostability of the formulations (Shibata et al., 2001).

### Pullulan derivative for improved pH sensitivity

A pH-sensitive material is a type of material that gets changed on alteration in pH of surroundings. It is a macro scoping property of the polymer. The pH-sensitive polymer can be served as a carrier for various drug delivery systems such as the controlled release of drug (by continuous pH change of gastrointestinal tract (GIT)) and targeted drug delivery (based on the difference between pH of normal tissue and infected tissue of the body) (Wang et al., 2020). There are two methods to improve the pH sensitivity of pullulan. This includes periodate oxidation and the formation of cationic pullulan.

An injectable pH-responsive and mucoadhesive hydrogel of doxorubicin were prepared for local drug delivery. Periodate oxidated pullulan was used with chitosan-grafted-dihydrocaffeic acid to formulate the hydrogel, which resulted in a pH-dependent effect with good injectability and quick gelation time. Another pH-sensitive hydrogel of amoxicillin was prepared using the same ingredient to inhibit the growth of *Escherichia coli* and *Staphylococcus aureus* (Liang et al., 2019).

Another way to enhance the pH sensitivity of pullulan is the formation of cationic pullulan. Cationic polymers are generally prepared for incredible encapsulation efficacy, improved bioavailability, low toxicity, and improved release profile. They are commonly used as drug delivery agents (Farshbaf et al., 2018). Cationic pullulan (DBAP-PO) can be prepared by reaction of pullulan with hydrophobic octyl and tertiary dibutylaminopropyl groups. The DBAP-PO is an amphiphilic pullulan derivative with positive charges, showing sustained and pH-dependent release with no cytotoxicity. A nanoparticle of diclofenac was prepared using cationic pullulan (DBAP-PO), which showed the pH-dependent release, sustained release effect, non-cytotoxicity, and enhanced safety. This indicated pullulan as a promising carrier for pH-responsive drug delivery to affected tissues (Constantin et al., 2020).

### Pullulan derivative for improved anticoagulant property

Anticoagulants are used to stop the thickening of the blood and the formation of clots. Heparin is one of the popular anticoagulants. Pullulan derivatization via sulfation has been done with the aim to develop an alternative to heparin. Sulfated pullulan can be obtained by the reaction of pullulan and sulfur trioxide–pyridine complex in DMF (dimethylformamide) at 75 °C and 95 °C for 3–8 h. Sulfate pullulan can also be obtained from pullulan reaction with SO_3_-DMF (*N*,*N*-dimethyl formamide) complex, but due to SO_3_–DMF complex, resultant pullulan becomes more reactive and less viscous; therefore, SO_3_–Py (pyridine) complex is more preferred to achieve stable and viscous derivative. Pullulan sulfate C-6 is the most preferential position for sulfation, followed by C-3, while C-4 remained mostly unsulfated (Mähner et al., 2001). It was reported that pullulan sulfate prevents coagulation by interfering with several stages of coagulation (Alban et al., 2002).

### Pullulan derivative for antimicrobial activity

Antimicrobials are the agent that prevents or stop the growth of micro-organism. Pullulan derivatives also can show antimicrobial action. CMP was prepared mainly using a crosslinking reaction with different diamines and dihydrazide. However, when pullulan is coprocessed with carboxymethyl chitosan, the resultant carboxymethyl chitosan pullulan film shows antimicrobial action (Li et al., 2011). This is because carboxymethyl chitosan has anti-microbial properties, which get adopted by pullulan during the reaction (Wu et al., 2013). A sponge was prepared using carboxymethyl chitosan for wound healing and dermal reconstruction, which showed great success in the form of sponge due to its high porosity, appropriate water vapor transmission rate, and swelling ability (Wang et al., 2017).

### Pullulan for improved drug delivery

Drug delivery is a method of delivering pharmaceutical compounds into the body to achieve a therapeutic effect (Tiwari et al., 2012). Since the exploration of diseases developed and biopharmaceutical achieved progress rapidly, conventional drug delivery systems cannot satisfy the demand anymore. Thus, the required novel drug delivery systems are liposomes, niosomes, proliposomes, microspheres, gels, prodrugs etc. Various types of material have been studied as carriers to explore their potential application in the formulation of novel drug delivery systems. Polysaccharides are one of the types among them, considering their biocompatibility and safety. Pullulan, an exopolysaccharide acts as a carrier due to its physicochemical properties. Modifying it further adds more properties, including stimuli-responsive ability, enhancing therapeutic efficiency, diagnosis, and releasing the combination therapy (Tong et al., 2020). For drug delivery, it can be modified via periodate oxidation, succinylation, cholesterol modification, sulfation, formation of cationic pullulan, incorporation of PEG, and carboxymethylation as listed in Table 2.

Periodate oxidation involves introducing the aldehyde group in pullulan, making pullulan capable of carrying macromolecules (Bruneel & Schacht, 1993). A scaffold was prepared using pullulan with collagen and loaded with lysine or hydroxylysine by freeze-drying method. The scaffold resulted in enhanced crosslinking due to the reaction of aldehyde of oxidated pullulan and amino group of lysine or hydroxylysine. The enhanced crosslinking decreases the rate of degradation. This indicated pullulan as promising bio-crosslinked for biomaterial synthesis (Selvakumar & Lonchin, 2020). Succinylation involves the introduction of a hydrophobic substance to pullulan, which can encapsulate hydrophobic and charged protein.

Pullulan undergoes carboxymethylation, which adds a negative charge. This product, in contrast to pullulan, has a low affinity for asialoglycoprotein receptors (Nogusa et al., 2000b). There was a more than 100-fold reduction in pullulan’s liver uptake clearance. The potential for using this derivative in chemotherapy was then explored. The researchers employed a peptide linker to attach doxorubicin, a well-known chemotherapy medication used to treat various tumors, to carboxymethylated pullulan. Macromolecular prodrugs are created by conjugating such small molecular drugs to polysaccharides, rendering them inert. To be pharmacologically effective, the conjugated drug needs to be released from the prodrug. Drug exposure to other vulnerable tissues and the free drug plasma levels are both decreased by conjugation. These prodrugs have a longer half-life than free drugs. This longer half-life causes prodrugs to passively accumulate in the tumor. This is because the tumor’s leaky vasculature increases the prodrug’s permeability to it, and the tumor’s reduced lymphatic outflow causes the macromolecular conjugates to be retained. In their in vivo trial, Nogusa et al. found that the conjugated drug was superior to the free drug in terms of efficacy. On murine carcinoma models, solid tumors and nonsolid tumors, they tested the conjugate and free medication. Compared to the free medication, the conjugation was more efficient at decreasing tumor volume and improving survival rates. The compound is solely effective against solid tumors (Nogusa et al., 2000a). Pullulan exhibited excellent property in increasing the drug release at the tumor site for Epirubicin, Adriamycin, Doxorubicin, Methotrexate, and Combretastatin as mentioned in Table 3.

**Table 3.** Enhanced drug delivery of anticancer drug using pullulan and derivatives.

Dosage Form	Drug	Polymer	Observations	References
Nanoparticle	Doxorubicin	Carboxymethyl Pullulan	After 21 h of incubation, it displayed a pH-dependent release characteristic, releasing 67% of the medication at pH 5.0 and 36% at pH 7.4.	(Lu et al., 2008)
		Aminated carboxymethyl pullulan	showed a pH-dependent release pattern, releasing 75% of the medication at pH 5.0 (2 h) and just 15% at pH 7.4. (12 h)	(Li et al., 2014)
		Folate-decorated maleilated pullulan	revealed a slow (>100 h) and pH-dependent (pH 5.0 > 7.4) release behavior, and the folate-adorned nanoparticles demonstrated improved cellular absorption.	(Li et al., 2013)
	Epirubicin	Cholesterol modified pullulan (CHP)	Showed enhanced *t*1/2 (19.33 h), Cmax (10.45 mg/L) and AUC (48.36 mg/L)	(Shen et al., 2014)
	Adriamycin	Pullulan acetate	Increased cytotoxicity to breast tumor cells at pH 6.8	(Na et al., 2003a)
		Vitamin H modified pullulan acetate	Increased the uptake of Adriamycin by HepG2 cells	(Na et al., 2003b)
		O-Urocanyl pullulan	Showed accelerated drug release (72.1%) at pH 5.8 (tumor cells)	(Guo et al., 2014)
	Mitoxantrone	Biotin modified cholesterol-pullulan	At pH 3.5, the medication was released from nanoparticles by over 90%. (tumor cells)	(Yang et al., 2014)
	Methotrexate and combretastatin A4	*N*-Urocanyl pullulan	Enhanced anticancer and antiangiogenic effects, and improved drug distribution in the liver and tumor	(Wang et al., 2013)
Hydrogel nanoparticles	Doxorubicin	Oligo (methacryloyl sulfadimethoxine) grafted pullulan acetate	After 12 h of incubation, drug release displayed pH-dependent patterns, with 85% of the drug released at pH 6.2 and 50% of the drug released at pH 7.4.	(Na et al., 2004)
Nanogel		Pullulan-*g*-poly(l-lactide)	The cumulative drug release was temperature-responsive, averaging about 40% at 25 °C, 65% at 37 °C, and 95% at 42 °C.	(Seo et al., 2012)

**Table 2.** Property enhancement due to derivatization of pullulan.

Improved property	Reaction	Substituted group	Drug loaded/ Substance	Design	Other polymer involved in formulation	Result	Reference
Pullulan derivative for enhanced solubility	Alkylation	Alkyl	–	–	–	Enhanced solubility in general organic solvent	(Shibata et al., 2002)
	Urethane derivative	phenylurethane and *N*-hexylurethane groups	–	–	–	Enhanced solubility in general organic solvent	(Shibata et al., 2001)
	Incorporation of PEG in pullulan	PEG	–	–	–	Enhanced solubility in Organic solvent.	(Jiao et al., 2004)
Pullulan derivative for improved mechanical property	Carboxymethylation		Mangoes	Edible coating film	Carboxymethyl chitosan and Galangal essential oil (GEO)	Edible coating film with better mechanical property, reduced cost and stronger antimicrobial property.	(Zhou et al., 2021)
	Carboxymethylation	acrylated carboxymethyl cellulose	Amoxycillin	Injectable hydrogel	Poloxamer 407	High elasticity of gel and sustain release effect of drug.	(Constantin et al., 2020)
	Periodate oxidation	CHO	–	Hydrotablegel	Gelatin	Enhanced mechanical property	(Wang et al., 2019)
Pullulan derivative for enhanced stability	Succinylation	carboxylic anhydrides	lysozyme	Microsphere	–	Long term stability and three time higher loading efficiency of protein as compare to PLGA microsphere.	(Woo et al., 2011)
	Succinylation	*n*-octenyl succinc anhydride	Strawberries	Edible Coating film	–	Enhanced shelf life of packed fruit.	(Niu et al., 2019)
	Cholesterol modified pullulan	Cholesterol	Epirubicin	Nanoparticle	–	Higher blood plasma concentration, longer half-life, reduced drug toxicity as compared to free Epirubicin.	(Shen et al., 2014)
	Cholesterol modified pullulan	Cholesterol	Mitoxantrone (MTO)	Nanoparticle	HSA	Targeted delivery of drug to tumor cell, enhance the efficacy, safety and stability.	(Yuan et al., 2019)
Pullulan derivative for improved pH sensitivity	Periodate oxidation of pullulan	CHO	Doxorubicin	Injectable hydrogel	chitosan-grafted-dihydrocaffeic acid	Good injectability, quick gelation time, in vitro pH-dependent equilibrated swelling ratios, interconnected morphologies, and good rheological properties with ability to kill colon tumor cell.	(Liang et al., 2019)
	Periodate oxidation of pullulan	CHO	Amoxicillin	Injectable hydrogel	chitosan-grafted-dihydrocaffeic acid	Inhibited the growth of E. coli and S. aureus in vitro.	(Liang et al., 2019)
	Cationic pullulan	DBAP	Diclofenac	Nanoparticle	–	Sustained and pH- dependent release.	(Constantin et al., 2020)
Pullulan derivative for improved anticoagulant property	Sulfation	SO_3_–pyridine complex	–	–	–	Anticoagulant effect	(Alban et al., 2002)
Pullulan derivative for antimicrobial activity	Carboxymethylation	acrylated carboxymethyl cellulose	–	Sponges	Carboxymethyl chitosan	Wound healing	(Wang et al., 2017)
Pullulan derivative for improved drug delivery	Periodate oxidation	CHO	lysine or hydroxylysine by freeze drying method.	Scaffold	collagen	Decreased the rate of degradation by improving crosslinking	(Selvakumar & Lonchin, 2020)
	Sulfation	SO_3_–pyridine complex	Model protein (BSA)	Nanoparticle for nasal and pulmonary route	–	Adequate size with no toxicity on delivering to respiratory cell.	(Dionísio et al., 2016)
	Cationic pullulan		MicroRNAs (miRNAs)	polyplexes	Quaternized ammonium group	Gene delivery inside the cell with no cytotoxicity	(Moraes et al., 2020)
	Carboxymethylation	acrylated carboxymethyl cellulose	Lysozyme	Microspheres	Palmitoyl	Controlled release of lysozyme which can be used for separation, purification or immobilization of the enzymes.	(Mocanu et al., 2004)
	Carboxymethylation	Acrylated carboxymethyl cellulose	Diphenhydramine (DPH)	Hydrogel	poly(*N*-isopropylacrylamide) (PNIPAAm).	pH and thermo-responsive sustain drug release.	(Asmarandei et al., 2013)
	Carboxymethylation	Acrylated carboxymethyl cellulose	Heparin	–	–	Inhibited the proliferation of Smooth muscle cells and proliferated vascular endothelial cells	(Na et al., 2003)

## Cholesterol-modified pullulan

**Figure 4.**
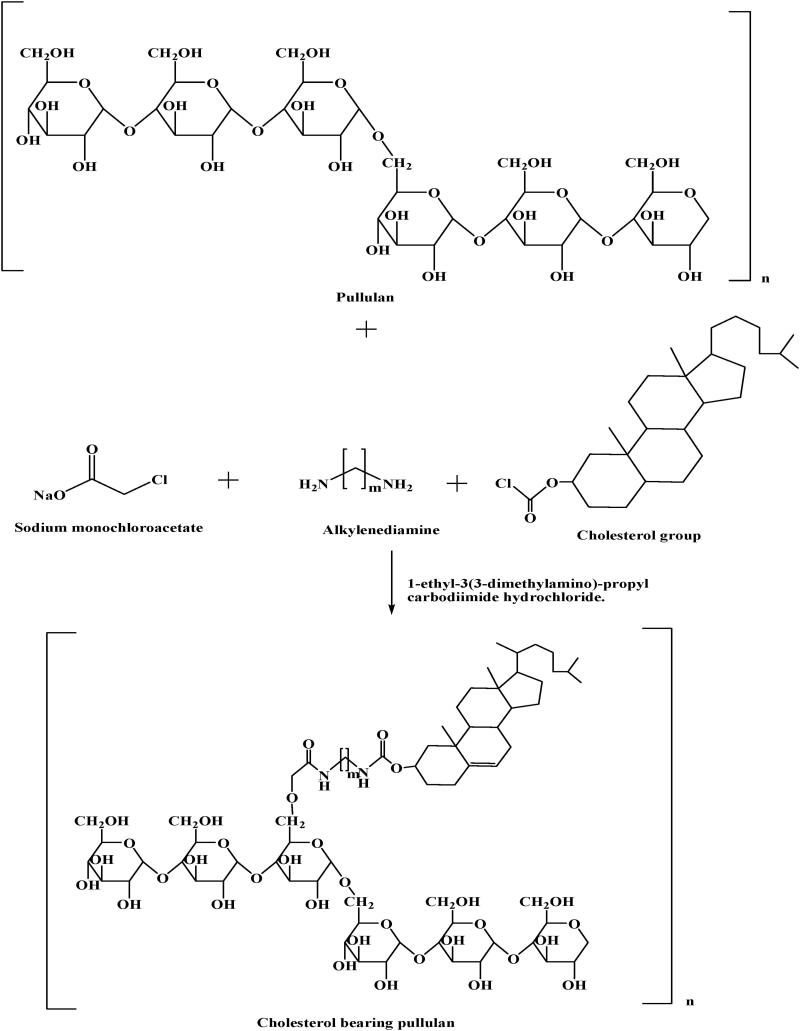
Synthetic scheme for cholesterol bearing pullulan.

Pullulan can be used to deliver antitumor drugs if it forms the self-assembled nanoparticle in the aqueous phase. For that, modification of pullulan is required by a hydrophobic group such as long carbon chains or cholesterol groups (Yuan et al., 2020). This will result in an amphiphilic polymer that can be processed to form the self-assembled nanoparticle. Pullulan with a hydrophobic group is prepared by binding of cholesterol group to pullulan via monochloroacetate and alkylenediamine in the presence of 1-ethyl-3(3-dimethylamino)-propyl carbodiimide hydrochloride as depicted in Figure 4 (Singh et al., 2015). Nanoparticles of cholesterol-modified pullulan showed higher loading capacity and sustained-release effect. The hydrophobicity of nanoparticles can be changed by altering the degree of substitution of cholesterol. Change in hydrophobicity will affect the size, drug loading, and release from the nanoparticle. Hydrophobicity is directly proportional to drug loading capacity, and drug release from nanoparticles i.e., higher the hydrophobicity, higher will be the drug loading and better sustain release of the drug. It is inversely proportional to the size of the nanoparticle that means an increase in hydrophilicity of pullulan will lead to a small size of the nanoparticle (Yuan et al., 2020).

Sulfation involves the introduction of sulfate trioxide into pullulan, which allows pullulan to deliver proteins (Mähner et al., 2001). A nanoparticle was prepared using sulfated pullulan to associate a model protein (BSA) upon polyelectrolyte complexation either carrageenan or chitosan. This nanoparticle showed adequate size and no toxicity on delivering protein on a respiratory cell line; therefore, pullulan with sulfate was found to be a good candidate for transmucosal protein delivery with special focus on nasal and pulmonary route (Dionísio et al., 2013).

The formation of cationic pullulan is another way to deliver drugs effectively due to the presence of positive charges (Constantin et al., 2020). A nanosized polyplex was prepared using cationic pullulan. For this, pullulan was first linked with the quaternized ammonium group, which further complexed with microRNAs (miRNAs). The microRNAs (miRNAs) complex with cationic pullulan successfully delivers the gene into the cell with no cytotoxicity and maintains aqueous stability. Thus, cationic pullulan can be used to deliver the gene (Moraes et al., 2020).

Carboxymethylation derivatization is one more way for the betterment of pullulan for drug delivery. It involves the introduction of carboxylate or carboxymethyl chitosan into the pullulan structure. A microsphere was prepared using carboxymethyl pullulan with palmitoyl hydrophobic group to load lysozyme. This resulted in the controlled release of lysozyme based on the content of carboxymethyl or palmitoyl group. The prepared microsphere can be used for purification, separation, or immobilization of the enzyme (Mocanu et al., 2004). A pH-sensitive hydrogel was prepared to deliver diphenhydramine (DPH) using carboxymethyl pullulan with thermosensitive and pH-sensitive polymer poly(*N*-isopropylacrylamide) (PNIPAAm). This combination resulted in thermo-responsive and pH-responsive hydrogel having sustained release effect at temperature 37 °C and pH 10. The drug release was highly influenced by temperature, pH, and composition of the hydrogel (Asmarandei et al., 2013).

Carboxylated pullulan was conjugated with heparin. By immobilizing heparin to pullulan, endothelial cells (ECs) attached to the heparin-conjugated pullulan were more aggregated than when attached to other pullulan derivatives and inhibited the proliferation of SMCs (smooth muscle cells) in vitro. Thus, carboxylated pullulan can be used to proliferate vascular ECs and inhibit the proliferation of SMCs (Na et al., 2003c).

## Application of pullulan

**Figure 5.**
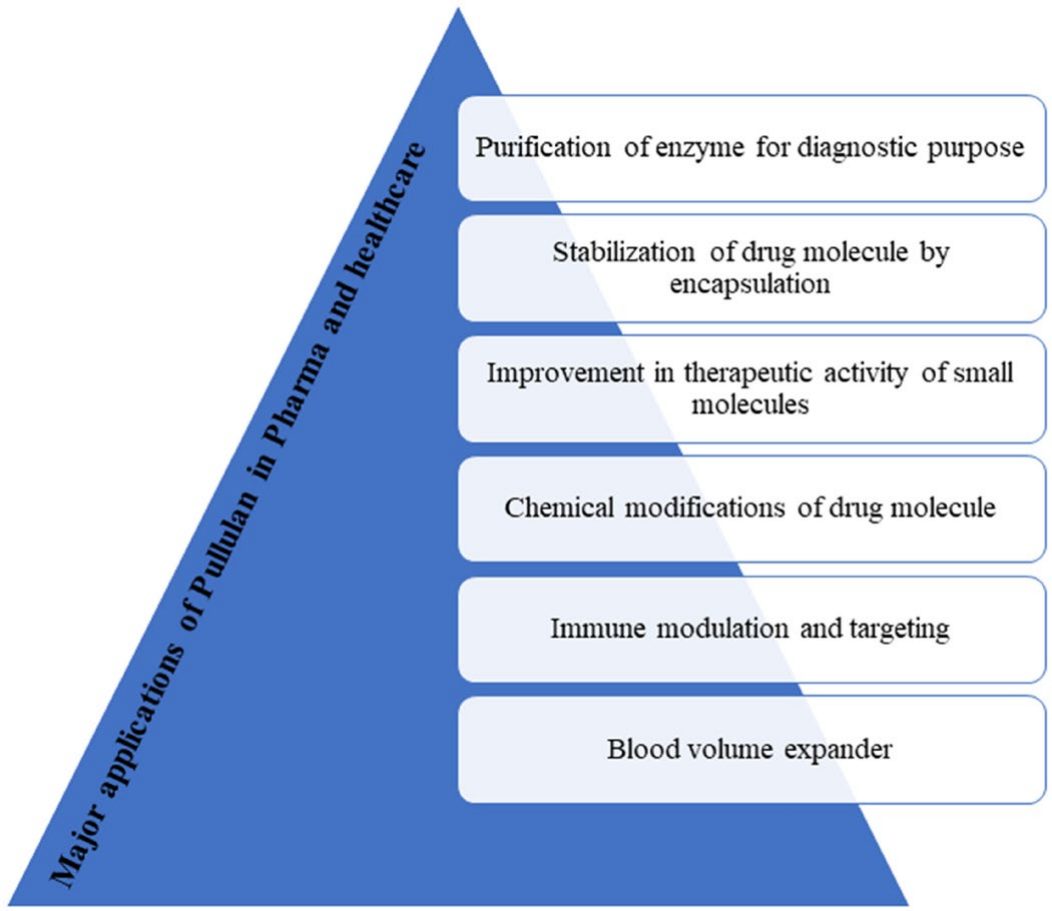
Major applications of pullulan in pharma and healthcare.

Pullulan has grabbed a lot of attention because of its unique characters. It has huge application in pharmaceutical industry as shown in Figure 5. Derivatization of pullulan via chemical reactions makes it more versatile. It can be used in coating of tablet, capsule, granules, pills, or topical formulation such as gel, film, etc. Pullulan capsules are suitable for sensitive nutraceutical like plant extracts. Patient compliance is more for pullulan capsule as it is vegetable capsule. An amoxicillin capsule using pullulan was prepared that showed extended release of amoxicillin in simulated gastric fluid with additional benefits like low water content, brittleness, leakage, and high tightness (Ding et al., 2020). Pullulan tablets exhibit better hardness, in vitro release, and maintain gastro resistance of microparticle (Patel et al., 2012). A tablet of sodium alendronate-loaded microparticles was prepared by direct compression using pullulan as filler (Ferreira et al., 2015). Its tissue engineering and grafting enhances its use in biomedical and pharmaceutical field. Also, it is widely used in targeted drug delivery (Tiwari et al., 2019). Pullulan gel has great potential in would healing (Thangavel et al., 2020). Pullulan film can be widely used in antibacterial packaging due to its oxygen and water vapor barrier property (Li et al., 2020). Pullulan fast dissolving oral strips are also available. Pullulan-based strips exhibits excellent content uniformity, immediate drug release of poorly water-soluble drug nanoparticles (Krull et al., 2016). A number of Marketed formulations are available using pullulan and few important formulations are listed in Table 4.

**Table 4.** Marketed preparations using pullulan.

Drug and dose	Formulation	Packager	Indication
Candesartan	Candesartan cilexetil tablet	Alembic Pharmaceuticals Inc.	Treatment of hypertension and heart failure
Paulinia cupana h.b.k et k	Eupepsia thin (paulinia cupana h.b et k) strip	Dalian Jixin Electronic and Communication Co., Ltd.	Homeopathic appetite suppresant
Salicylic acid	Age bright spot fader (salicylic acid) gel and cream	Dermalogica, Inc. & Nerium Skincare, Inc.	Controls and helps prevent recurrence of skin itching, irritation, redness, flaking and scaling associated with seborrheic dermatitis, and or psoriasis
Laburnum anagyroides 1×	Tbx-free- laburnum anagyroides strip	Advanced Men’s Institute LLC	Homeopathic formulation for treatment of chronic nicotinism
Riluzole 50 mg	Exservan (riluzole) film	Mitsubishi Tanabe Pharma America, Inc	Treatment of amyotrophic lateral sclerosis
Camphor 3%	Neriumrx dermal pain therapy (camphor 3%) spray	Nerium Skincare, Inc.	Temporary relief of pain and itching.
Loratadine 10 mg	Allervarx (loratadine) tablet, orally disintegrating	Innovus Pharmaceuticals, Inc. & Strides Pharma Inc	Relieves these symptoms due to hay fever or other upper respiratory allergies
Boric acid	V-bella (boric acid) suppository	US Pharmaceutical Corporation	Restores vaginal pH and relieves vaginal itching, burning and irritation
Niacinamide (2%)	Varecell Intensive liposome doublecream (niacinamide, adenosine, aluminum sucrose octasulfate) cream	D&S Cosmedique Co. Ltd.	Prevent wrinkles and brighten skin
Aluminum sucrose octasulfate (0.18%)			
Adenosine (0.04%)			

## Prospects

Pullulan is a biopolymer with excellent properties. To enhance its scope, chemical derivatization has been done, which resulted in improved solubility of pullulan in the organic solvent, improved mechanical property, pH sensitivity, better drug and gene delivery, anticoagulant, and antimicrobial property. The application of modern technologies, such as nanoparticles, nanogels, liposomes, microspheres, scaffolds with grafted pullulan and thin films for gene delivery, immunization, and cosmetics, has to be explored in greater detail. Further work should be done on formulation using alkyl pullulan, sulfated pullulan, urethane derivative, and pullulan-PEG in order to prove the effectiveness of these derivatives. Also, with the additional research, the physical and chemical properties of pullulan can be continuously improved to obtain a more suitable derivative for medical use and drug delivery systems. Although lots of marketed formulations are available with pullulan and lots of research is available on pullulan derivatives, the pullulan derivatives are still under investigation and yet to be approved for commercial use.

## Conclusion

Research studies have revealed pullulan as a unique exopolysaccharide with a variety of potential industrial and medical applications. It has properties like suitable adhesion, anti-oxidant, film-forming, blood compatibility, biodegradability, mucoadhesion, etc. Despite having a large number of valuable properties, its insolubility in the organic solvent, poor mechanical property, and unavailability of functional groups to carry macromolecules limit its application in pharmaceutical fields. However, the pullulan structure has nine hydroxyl groups liable to the chemical reaction. Replacement of hydroxyl group with alkyl, *N*-phenyl urethane, *N*-hexyl urethane groups results in improved solubility of pullulan in organic solvents, replacement of hydroxyl with carboxymethyl or aldehyde results in improved mechanical property, replacement of hydroxyl group with the carboxylic group, cholesterol, *N*-phenyl urethane or *N*-hexyl urethane results in improved stability, replacement of hydroxyl group with aldehyde or DBAP results in proved pH sensitivity, replacement of hydroxyl group with aldehyde, cholesterol, sulfur trioxide, DBAP, or carboxymethyl results in improved drug delivery systems, replacement of hydroxyl group with sulfur trioxide and carboxymethyl results in improved anticoagulant and antimicrobial property respectively.
